# Impact of an assessment form on promoting in-hospital initiation of sodium–glucose cotransporter-2 inhibitors in hospitalized patients with heart failure

**DOI:** 10.1016/j.ijcrp.2026.200577

**Published:** 2026-01-08

**Authors:** Takuya Okamoto, Koichiro Matsumura, Hiroyo Miyata, Yuta Kimoto, Kazue Hamamura, Keiko Kato, Shohei Hakozaki, Eijiro Yagi, Masafumi Ueno, Kimiko Fujiwara, Manabu Takegami, Gaku Nakazawa

**Affiliations:** a Department of Pharmacy, Kindai University Hospital, Sakai, Japan; b Department of Cardiology, Kindai University Faculty of Medicine, Sakai, Japan; c Department of Clinical Nutrition, Kindai University Hospital, Sakai, Japan; d Department of Rehabilitation, Kindai University Hospital, Sakai, Japan; e Department of Nursing, Kindai University Hospital, Sakai, Japan

**Keywords:** SGLT2 inhibitors, Heart failure, Guideline-directed medical therapy

## Abstract

**Background:**

Despite the established benefits of in-hospital administration of sodium–glucose cotransporter-2 inhibitors (SGLT2i) in patients with heart failure (HF), their implementation remains insufficient. To investigate whether the use of an Assessment Form influences SGLT2i prescriptions at discharge among hospitalized patients with HF.

**Methods:**

We retrospectively analyzed consecutive patients with HF from a prospective registry between September 2024 and August 2025 after the implementation of an Assessment Form. Patients who died during hospitalization were excluded. The Assessment Form was completed collaboratively by physicians and nurses. Physicians checked whether guideline-directed medical therapy (including SGLT2i) had been prescribed, and documented the reasons if not initiated, while nurses evaluated the patients’ pre-hospital living conditions and self-care abilities. The primary endpoint was the prescription rate of SGLT2i at discharge according to the Assessment Form.

**Results:**

Among the 208 analyzed patients (median age, 81 years [range, 75–87] years, 59 % male], the Assessment Form was used by 61.1 % (127/208). The prescription rate of SGLT2i at discharge was significantly higher in patients who completed the Assessment Form than in those who did not (65.4 % vs. 45.7 %, *p* < 0.01). Multivariable logistic regression identified use of the Assessment Form as an independent factor associated with SGLT2i prescription at discharge (odds ratio 2.12, 95 % confidence interval 1.04–4.32, *p* = 0.03).

**Conclusion:**

Implementation of an Assessment Form was associated with increased initiation of in-hospital SGLT2i therapy. The active use of such structured forms may help promote adherence to guideline-directed therapies during hospitalization for HF.

## Highlights

• SGLT2i initiation in hospitalized patients with HF is important.
• Despite this, the use of SGLT2i remains suboptimal in real-world practice.
• An assessment form may promote SGLT2i use in hospitalized patients with HF.
• The assessment form increased initiation of in-hospital SGLT2i therapy.
• Use of such forms may help promote adherence to guideline-directed therapies.

## 1 Introduction

Heart failure (HF) is a chronic progressive condition that continues to increase in prevalence with population aging, leading to frequent hospital readmissions and a marked decline in patients’ quality of life [1]. Recent advances in HF management have renewed the importance of guideline-directed medical therapy (GDMT) [2]. Among these therapies, sodium–glucose cotransporter-2 inhibitors (SGLT2i) have emerged as one of the four foundational pillars of HF treatment, demonstrating benefits in reducing mortality and hospital readmissions regardless of ejection fraction phenotype [3]. SGLT2i are characterized by minimal worse effects on renal function and blood pressure, and their clinical efficacy becomes apparent shortly after initiation [4].

Introducing SGLT2i during HF hospitalization is considered a rational strategy to prevent early post-discharge decompensation [5]. Nevertheless, the actual prescription rate of SGLT2i in clinical practice remains suboptimal, particularly among older adults and patients with multiple comorbidities [6,7]. Various factors, such as concerns about renal function and blood pressure, polypharmacy, financial burden, uncertainty about prescribing responsibility, and limited awareness among healthcare providers, are thought to contribute to this gap in implementation [3]. Accordingly, there is a growing need for practical strategies to promote the initiation of SGLT2i in hospitalized patients [8].

Previous approaches have included physician education programs, the establishment of institutional protocols, and pharmacist-led interventions; however, simple and feasible mechanisms applicable in routine clinical settings are still lacking [9,10]. In particular, few studies have evaluated checklist-based interventions that enable multidisciplinary teams to confirm therapy initiation and document reasons for non-prescription [11,12]. Recently, the use of structured checklists has been shown to improve patient safety and adherence to standard care across multiple clinical domains, also suggesting potential applicability to HF management [13]. Therefore, in this study, we introduced an Assessment Form jointly completed by physicians and nurses, and investigated its impact on the prescription rate of SGLT2i at hospital discharge among patients hospitalized for HF.

## 2 Methods

### 2.1 Study design and population

**Fig. 1:**
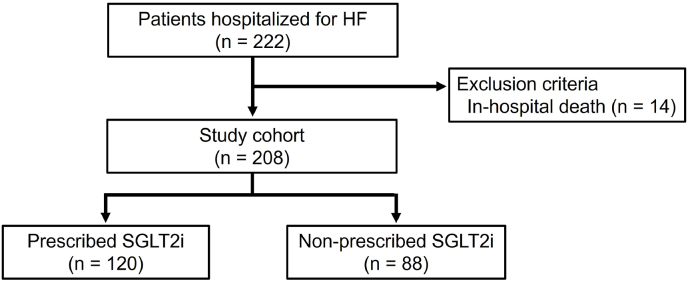
**Study flowchart** HF: Heart failure, SGLT2i: Sodium–glucose cotransporter-2 inhibitor.

This single-center, retrospective observational study was conducted at our hospital. Data were obtained from the registry data, a prospective registry that enrolled hospitalized patients with HF. The registry included patients aged ≥20 years who were admitted with a primary diagnosis of acute decompensated HF. Patients with HF secondary to acute coronary syndrome, those receiving maintenance dialysis, those with HF associated with severe aortic stenosis, or those with pulmonary hypertension as the primary pathology were excluded. For the present analysis, we included consecutive patients enrolled in the registry between September 2024 and August 2025, which corresponds to the period after the implementation of the assessment form at our institution. Among the 222 eligible patients, those who died during hospitalization (n = 14) were excluded, leaving the remaining patients for the analysis (Fig. 1). Patient consent was obtained using an opt-out approach. The study protocol was approved by the Ethics Committee of our hospital and was conducted in accordance with the principles outlined in the Declaration of Helsinki.

### 2.2 Data collection

Baseline demographic and clinical data, including age, sex, body mass index, New York Heart Association functional class, blood pressure, and heart rate at admission, were extracted from electronic medical records. Information regarding pre-admission comorbidities and medications was obtained from the electronic medical systems. Laboratory data were collected upon admission, and the geriatric nutritional risk index (GNRI) was calculated using serum albumin levels and body mass index as indicators of nutritional status. The left ventricular ejection fraction (LVEF) was assessed using transthoracic echocardiography performed during hospitalization. From electronic records, we confirmed that each patient was prescribed an SGLT2i at discharge. In addition, the prescription status of other GDMT, including angiotensin-converting enzyme inhibitors (ACEi), angiotensin receptor blockers (ARB), angiotensin receptor–neprilysin inhibitors (ARNI), beta-blockers, and mineralocorticoid receptor antagonists (MRA), was also reviewed at discharge.

### 2.3 Assessment form

The Assessment Form used in this study was an electronic form integrated into the hospital's medical records system and consisted of sections completed by both physicians and nurses. Physicians evaluated whether the four components of GDMT, including SGLT2i, were initiated for each patient and indicated this by marking the corresponding items on the form. When no therapy was initiated, the reason was recorded by the physician. Nurses interviewed patients and their family members to obtain the following information: management of medication before admission, methods of medication management, adherence to medication, preparation of meals, attention to salt restriction, approximate daily fluid intake, routine blood pressure monitoring, availability of a blood pressure monitor and a scale at home, exercise habits, employment status, financial concerns, perceived burden of outpatient visits after discharge, smoking and drinking habits, and family support in daily life. Based on these findings, nurses provided education to patients and their families on the importance of HF self-management in accordance with current HF guidelines and explained how to use the HF handbook in daily practice [14]. Physicians reviewed the information collected by nurses through the Assessment Form to guide decision-making regarding post-discharge management. Completion of the electronic Assessment Form was not mandatory, and was incorporated into routine clinical practice after its implementation. Whether the Assessment Form was completed for a given patient was determined by the attending physicians and nursing staff based on the clinical circumstances and workflow considerations during hospitalization.

### 2.4 Outcome measures

The primary outcome was the prescription rate of SGLT2i at discharge, which was compared between patients with and without completion of the Assessment Form during hospitalization. Secondary outcomes included the prescription rates of ACEi, ARB, ARNI, MRA, and beta-blockers at discharge, according to the Assessment Form. The analysis of beta-blocker prescriptions was limited to patients with an LVEF <50 %.

### 2.5 Statistical analyses

Continuous variables are expressed as medians with interquartile ranges, and categorical variables are presented as counts and percentages. Differences between groups were assessed using the Wilcoxon rank-sum test for continuous variables and the chi-squared test for categorical variables. Logistic regression analyses were performed to determine whether use of the Assessment Form was independently associated with SGLT2i initiation. Variables with a *p*-value <0.05 in baseline characteristics between patients with and without prescriptions of SGLT2i at discharge were included in the univariate and multivariate analyses. A two-sided *p*-value <0.05 was considered statistically significant for all analyses. Statistical analyses were conducted using JMP software, version 18.2.0 (SAS Institute Inc., Cary, NC, USA).

## 3 Results

Of the 208 patients included in the analysis (median age 81 years [interquartile range 75–87 years], 59 % male), 61.1 % (127/208) used the Assessment Form during hospitalization. When the study period was divided into two phases, the proportion of patients for whom the Assessment Form was used did not differ between the first half of the study period (September 2024 to February 2025: 61.3 %) and the second half (March 2025 to August 2025: 60.7 %) (p = 0.92), indicating no significant temporal change in the utilization of the Assessment Form during the study period.

### 3.1 Patient characteristics

Patients who were prescribed SGLT2i at discharge (n = 120) were younger, had a higher proportion of males, a higher body mass index, and a higher prevalence of dyslipidemia than did those who were not prescribed SGLT2i (n = 88) (Table 1). The New York Heart Association functional class, blood pressure, and heart rate at admission were similar between the two groups. The prevalence of prior hospitalization for HF, myocardial infarction, or malignancy did not differ significantly between the groups. The proportion of patients who were already on SGLT2i prior to admission was significantly higher in the patients with prescribed SGLT2i (45 % vs. 9 %, p < 0.0001). In contrast, there were no significant differences in the proportion of patients receiving other HF medications at the time of admission between the groups.

**Table 1:** Patient characteristics at admission.

	Prescribed SGLT2i (n = 120)	Non-prescribed SGLT2i (n = 88)	*p*-value
Age (years)	80.0 [72.3–85.0]	83.0 [77.3–89.0]	0.001
Male	80 (67)	42 (48)	<0.01
BMI (kg/m^2^)	21.7 [19.1–24.4]	19.8 [18.2–22.4]	<0.01
NYHA class III/IV	112 (93)	83 (94)	0.77
Systolic blood pressure (mm Hg)	141 [119–164]	139 [129–166]	0.40
Diastolic blood pressure (mm Hg)	83 [70–97]	83 [70–100]	0.88
Heart rate (bpm)	92 [77–115]	89 [75–110]	0.65
Comorbidity			
Hypertension	98 (82)	72 (82)	0.98
Dyslipidemia	74 (62)	40 (45)	0.02
Diabetes mellitus	48 (40)	27 (31)	0.17
Atrial fibrillation	57 (48)	39 (44)	0.65
Prior HF hospitalization	37 (31)	18 (20)	0.09
Prior myocardial infarction	41 (34)	27 (31)	0.60
Malignancy	15 (13)	5 (6)	0.10
Prehospital medication			
Loop diuretics	58 (48)	39 (44)	0.57
ACEi/ARB/ARNI	66 (55)	46 (52)	0.70
Beta-blocker	61 (51)	33 (38)	0.06
MRA	37 (31)	22 (25)	0.36
SGLT2i	54 (45)	8 (9)	<0.0001

Data are presented as median (25th to 75th percentiles) or numbers (%).

ACEi: Angiotensin-converting enzyme inhibitor, ARB: Angiotensin II receptor blocker, ARNI: Angiotensin receptor–neprilysin inhibitor, BMI: Body mass index, HF: Heart failure, MRA: Mineralocorticoid receptor antagonist, NYHA: New York Heart Association, SGLT2i: Sodium–glucose cotransporter-2 inhibitor.

Patients who were prescribed SGLT2i had significantly higher hemoglobin and serum albumin levels at admission than did those who were not prescribed SGLT2i (Table 2). In addition, high-sensitivity C-reactive protein levels and LVEF were significantly lower in patients prescribed SGLT2i. The GNRI, a marker of nutritional status, was significantly higher in patients who were prescribed SGLT2i than in those who were not. No significant differences were observed in renal function, hemoglobin A1c, or B-type natriuretic peptide levels between the two groups.

**Table 2:** Clinical parameters at admission.

	Prescribed SGLT2i (n = 120)	Non-prescribed SGLT2i (n = 88)	*p*-value
Laboratory parameters			
Hemoglobin (g/dL)	12.3 [10.8–13.8]	11.2 [9.3–12.4]	<0.001
eGFR, (mL/min/1.73m^2^)	46 [33–61]	44 [27–62]	0.42
Serum albumin (g/dL)	3.6 [3.3–3.9]	3.4 [3.0–3.7]	0.001
High-sensitive CRP (mg/dL)	0.52 [0.24–1.92]	1.04 [0.41–3.77]	0.01
HbA1c (%)	6.2 [5.7–6.8]	6.0 [5.5–7.0]	0.13
BNP (pg/mL)	746 [380–1489]	743 [339–1300]	0.82
GNRI	94.9 [88.6–103.3]	89.2 [82.3–95.9]	<0.0001
LVEF (%)	44 [30–59]	50 [39–60]	0.04

Data are presented as median (25th to 75th percentiles) or numbers (%).

BNP: B-type natriuretic peptide, CRP: C-reactive protein, eGFR: Estimated glomerular filtration rate, GNRI: Geriatric nutritional risk index, HbA1c: Hemoglobin A1c, LVEF: Left ventricular ejection fraction.

### 3.2 Prescription rate of SGLT2i and other GDMT at discharge according to assessment Form

**Fig. 2:**
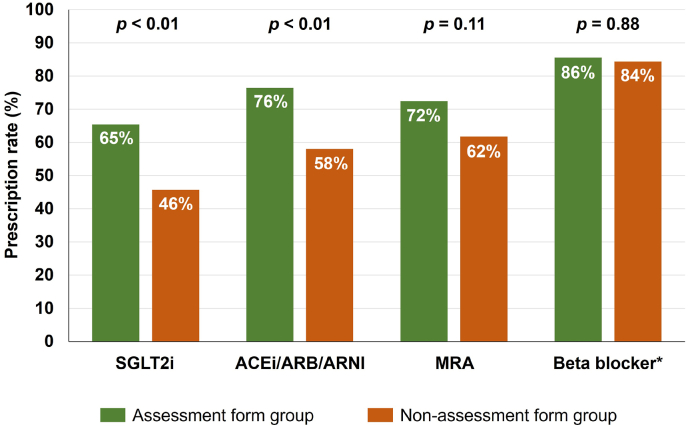
**Comparison of prescription rate of SGLT2i and other guideline-directed medical therapy at discharge according to use of assessment form** ∗ Analyzed for patients with LVEF <50 %. ACEi: Angiotensin-converting enzyme inhibitor, ARB: Angiotensin II receptor blocker, ARNI: Angiotensin receptor–neprilysin inhibitor, LVEF: Left ventricular ejection fraction, MRA: Mineralocorticoid receptor antagonist, SGLT2i: Sodium–glucose cotransporter-2 inhibitor.

The prescription rate of SGLT2i at discharge was significantly higher in the Assessment Form group than in the non-Assessment Form group [65.4 % (83/127 patients) vs. 45.7 % (37/81 patients), p < 0.01, Fig. 2]. Similarly, the prescription rate of ACEi, ARB, or ARNI at discharge was significantly higher in the Assessment Form group [76.4 % (97/127 patients) vs. 58.0 % (47/81 patients), p < 0.01]. In contrast, there was no significant difference in the prescription rate of MRA between the two groups [Assessment Form group: 72.4 % (92/127 patients) vs. non-Assessment Form group: 61.7 % (50/81 patients), p = 0.11]. Among patients with LVEF <50 %, the prescription rate of beta-blockers at discharge was similar between the two groups [Assessment Form group: 85.5 % (65/76 patients) vs. non-Assessment Form group: 84.4 % (27/32 patients), p = 0.88].

### 3.3 Factors associated with prescribed SGLT2i at discharge

To evaluate whether the use of the Assessment Form was associated with prescribed SGLT2i at discharge, logistic regression analysis was performed (Table 3). Patient characteristics that differed significantly between those who were prescribed SGLT2i at discharge and those who were not were entered into a univariable analysis. The results demonstrated significant associations for all factors except the hemoglobin level at admission. Multivariate logistic regression analysis revealed that the use of the Assessment Form was independently associated with SGLT2i prescription at discharge (odds ratio 2.16, 95 % confidence interval 1.06–4.40, p = 0.03). Additionally, prehospital SGLT2i prescription and high-sensitivity C-reactive protein levels were significantly associated with prescribed SGLT2i at discharge.

**Table 3:** Logistic regression analysis of factors associated with prescribed SGLT2i at discharge.

	Univariable		Multivariable	
	OR (95 % CI)	*p*-value	OR (95 % CI)	*p*-value
Age (years)	0.95 (0.92–0.98)	<0.001	0.96 (0.93–1.00)	0.07
Male	2.19 (1.25–3.87)	<0.01	1.34 (0.67–2.69)	0.41
Dyslipidemia	1.93 (1.11–3.39)	0.02	1.58 (0.80–3.13)	0.19
Prehospital SGLT2i prescription	8.18 (3.82–19.70)	<0.0001	8.92 (3.53–22.52)	<0.0001
Hemoglobin (g/dL)	1.01 (0.96–1.10)	0.62	0.98 (0.88–1.04)	0.65
High-sensitive CRP (mg/dL)	0.88 (0.79–0.98)	0.01	0.86 (0.75–0.98)	0.03
GNRI	1.05 (1.02–1.08)	<0.001	1.02 (0.99–1.06)	0.18
LVEF	0.98 (0.96–1.00)	0.03	0.99 (0.97–1.02)	0.66
Assessment form	2.24 (1.27–3.99)	<0.01	2.16 (1.06–4.40)	0.03

CI: Confidence interval, CRP: C-reactive protein, GNRI: Geriatric nutritional risk index, LVEF: Left ventricular ejection fraction, OR: Odds ratio, SGLT2i: Sodium–glucose cotransporter-2 inhibitor.

## 4 Discussion

This study examined whether the use of an Assessment Form by physicians and nurses contributes to the promotion of SGLT2i initiation during hospitalization for heart failure. The results demonstrated that patients for whom the Assessment Form was used had a significantly higher rate of SGLT2i initiation. Furthermore, after adjusting for confounding factors, use of the Assessment Form was independently associated with SGLT2i use at discharge. These findings suggest that interventions using heart failure checklist may be effective in promoting SGLT2i initiation in real-world clinical practice.

The initiation of SGLT2i in hospitalized patients for HF plays a crucial role in improving both disease status and prognosis [15]. Patients who did not receive SGLT2i prior to discharge have been reported to have higher risks of HF readmission and cardiovascular death, highlighting the importance of early intervention [16]. However, in real-world practice, in-hospital initiation of SGLT2i remains suboptimal, and many patients do not receive these agents by the time of discharge [17]. Delayed initiation is influenced by multiple factors, including impaired renal function, hypotension, risk of dehydration, polypharmacy, cost burden, and delays or lack of awareness on the part of healthcare providers [8]. These factors hinder optimal medication management during hospitalization, potentially leading to increased post-discharge development of cardiovascular events. Moreover, the initiation of medication at discharge is closely linked to subsequent outpatient management and patient self-care. Insufficient GDMT initiation during hospitalization may negatively affect patient adherence to treatment and prognosis after discharge. Therefore, systematic and practical interventions are needed to ensure appropriate in-hospital initiation of GDMT, including SGLT2i, in patients with HF.

Several previous studies have reported the effectiveness of checklist-based interventions in promoting GDMT initiation in hospitalized patients with HF. Basoor et al. implemented a pre-discharge checklist to evaluate GDMT prescription status and reported that the prescription rates of ACEi or ARBs and beta-blockers improved by approximately 15 %–30 % [11]. Their study emphasized that the checklist functioned as a practical “reminder” tool for physicians to optimize therapy. However, initiation of SGLT2i was not evaluated in that study. Rismiati et al. also demonstrated that the introduction of a discharge checklist was associated with improved GDMT achievement rates and better one-year composite outcomes [12]. However, in their study, the checklist did not significantly increase the prescription rate of GDMT except for beta-blockers, whereas number of prescribed GDMT at discharge increased. These previous studies primarily focused on traditional GDMT components, such as ACEi/ARB, beta-blockers, and MRA, and to date, no study has specifically examined checklist-based interventions targeting SGLT2i initiation. In this context, our study is unique in demonstrating that the use of an Assessment Form significantly improved the rate of SGLT2i prescriptions at discharge. Moreover, while the improvement in medication optimization reported by Basoor et al. mainly relied on physician-driven processes, our intervention involved both physicians and nurses through a collaborative Assessment Form [11]. This multidisciplinary approach may represent a more comprehensive strategy for optimizing in-hospital management. The development of assessment tools that facilitate the involvement of multiple healthcare professionals could further enhance integrated patient care, including pharmacological interventions.

The Assessment Form used in this study may not only facilitate the initiation of SGLT2i during hospitalization, but also serve as a foundation for broader digital health strategies aimed at enhancing long-term treatment adherence and patient engagement. Digital health interventions, including mobile health applications, remote monitoring, and technology-enabled follow-ups, have been reported to improve self-management behaviors, medication adherence, and even clinical outcomes in patients with cardiovascular diseases, particularly those with acute coronary syndromes [18]. Therefore, integrating structured assessment tools with digital platforms may help sustain the benefits of in-hospital optimization during the post-discharge period and support the continuity of evidence-based pharmacotherapy. Importantly, the clinical significance of promoting the early initiation of SGLT2i extends beyond guideline adherence alone. Recent meta-analyses have demonstrated that SGLT2i significantly improve global cardiac structure and function, underscoring their physiological benefits [19]. These improvements are clinically relevant and suggest that timely in-hospital initiation of SGLT2i should be regarded not merely as a process-of-care indicator but as a substantive therapeutic intervention directly linked to improved cardiac function and long-term prognosis [20]. From this perspective, the Assessment Form represents a pragmatic tool that bridges evidence-based pharmacotherapy with real-world clinical implementation. Its clinical impact may be further amplified when combined with digital health-supported adherence strategies.

The Assessment Form implemented in this study represents a multicomponent intervention that integrates physician decision support with detailed nurse-led assessments and patient education rather than a simple checklist. While this comprehensive approach reflects real-world clinical practice and may have enhanced overall effectiveness, its generalizability may be limited to other institutions with different staffing resources, care structures, or levels of multidisciplinary collaboration. In addition, because multiple components were introduced simultaneously, it was difficult to disentangle the relative contribution of each element to the observed increase in SGLT2 inhibitor prescription rates. Future studies using stepwise implementation strategies or comparative study designs are required to identify the most effective components and further evaluate the external validity of these findings.

In our study, several potential mechanisms may explain how the use of the Assessment Form contributed to the initiation of SGLT2i. First, the Assessment Form provided physicians with a structured opportunity to systematically review the status of SGLT2i initiation before discharge, thereby reducing the likelihood of omissions. Second, by documenting the specific reasons for noninitiation, the form facilitated information sharing among physicians and enhanced communication within multidisciplinary teams. Third, nurses' assessment of patients' and families’ self-care capacities and social backgrounds may have helped identify those likely to maintain adherence after discharge, thereby creating a supportive environment that enabled physicians to initiate new medications with greater confidence. Patolia et al. highlighted multidisciplinary collaboration, comprehensive pre-discharge evaluation, and electronic checklist-based approaches as key strategies to promote the implementation of GDMT in HF care [8]. Our findings are consistent with and further support these practical implementation strategies.

## 5 Strength and limitations

Our study demonstrated that the use of an Assessment Form-based tool significantly improved the implementation rate of SGLT2i as part of GDMT in patients hospitalized for HF. Delayed in-hospital initiation of SGLT2i remains a major factor contributing to missed clinical benefits, underscoring the need to overcome physician-related clinical inertia [15]. Despite strong evidence supporting their early use, treatment initiation is often postponed in real-world practice. From this perspective, checklist-based interventions can function as “behavioral prompts,” allowing physicians and allied healthcare professionals to visualize and actively address treatment gaps. Moreover, the Assessment Form enables clinicians to explicitly document the reasons for noninitiation, thereby identifying barriers to optimal pharmacotherapy and providing opportunities to address them before discharge. By structuring this process, the tool promotes an environment in which clinicians can proactively review and adjust treatment strategies, potentially mitigating delays in the initiation of SGLT2i. Importantly, previous studies have shown that early in-hospital initiation of SGLT2i can lead to short-term improvements in clinical outcomes, emphasizing the importance of timely implementation [5]. Our findings suggest that the Assessment Form may serve as a practical approach to overcoming clinical inertia and could be further enhanced through multidisciplinary collaboration and multicenter application. In addition, the Assessment Form is simple to introduce in clinical settings as it is integrated within the electronic medical record system and requires no special equipment or additional staffing. Because both physicians and nurses can participate in its use, it represents a “low-burden, multidisciplinary” intervention that may serve as a feasible and scalable strategy to promote SGLT2i initiation in routine clinical care.

Nevertheless, this study has some limitations. First, this was a single-centre, retrospective, non-randomized observational study; therefore, causal relationships between use of the assessment form and SGLT2 inhibitor prescription cannot be definitively established. In addition, because the study was conducted over nearly one year, temporal trends such as increasing awareness of guideline-recommended SGLT2 inhibitor therapy, as well as concurrent institutional or quality-improvement initiatives, may have contributed to the observed increase in prescribing independent of the Assessment Form. Although the utilization of the Assessment Form itself remained stable over time, the absence of a formal time-trend analysis for prescribing behavior limits the ability to fully disentangle the effect of the intervention from secular changes in clinical practice. Second, the implementation of the Assessment Form may have been influenced by factors such as physicians' experience levels or team structure, leaving room for unmeasured confounders. In addition, the completion of the Assessment Form was not mandatory and was left to routine clinical practice, depending on clinical circumstances and workflow, resulting in unequal exposure to the intervention. Consequently, the patients for whom the form was completed may have differed systematically from those who were not exposed to the intervention, and the potential influence of selection bias cannot be completely excluded. Third, decisions regarding SGLT2i initiation likely depend on the physicians’ clinical judgment and patient preferences, leading to potential variability in prescribing behaviors. Fourth, the sample size was insufficient to adequately evaluate the impact of the Assessment Form on other GDMT components such as ACEi/ARB/ARNI, MRA, or beta-blockers. Despite these limitations, our study provides clinically meaningful evidence that a simple pre-discharge Assessment Form can facilitate the prescribed SGLT2i among hospitalized patients with HF. Future multicenter and prospective interventional studies are warranted to determine whether the implementation of such tools can improve the overall achievement of GDMT and long-term clinical outcomes. Furthermore, integrating pharmacists, physical therapists, and other healthcare professionals into the assessment form-based approach may help establish a more sustainable and systematic strategy for comprehensive GDMT implementation.

## 6 Conclusions

This study demonstrated that the implementation of an Assessment Form completed jointly by physicians and nurses effectively facilitated the prescribed SGLT2i as part of GDMT in patients hospitalized for HF. The use of this tool may help clarify the reasons for noninitiation and contribute to overcoming treatment delays in real-world clinical practice.

## CRediT authorship contribution statement

**Takuya Okamoto:** Writing – original draft, Methodology, Investigation, Formal analysis, Data curation, Conceptualization. **Koichiro Matsumura:** Writing – review & editing, Writing – original draft, Supervision, Methodology, Conceptualization. **Hiroyo Miyata:** Methodology, Formal analysis, Data curation. **Yuta Kimoto:** Methodology, Formal analysis, Data curation. **Kazue Hamamura:** Methodology, Formal analysis, Data curation. **Keiko Kato:** Methodology, Formal analysis, Data curation. **Shohei Hakozaki:** Methodology, Formal analysis, Data curation. **Eijiro Yagi:** Methodology, Formal analysis, Data curation. **Masafumi Ueno:** Writing – original draft, Methodology, Formal analysis, Conceptualization. **Kimiko Fujiwara:** Visualization, Methodology, Investigation, Formal analysis, Conceptualization. **Manabu Takegami:** Writing – original draft, Supervision, Methodology, Investigation, Conceptualization. **Gaku Nakazawa:** Writing – review & editing, Supervision, Methodology, Conceptualization.

## Funding statement

This research did not receive any specific grant from funding agencies in the public, commercial, or not-for-profit sectors.

## Declaration of competing interest

The authors declare no conflicts of interest.

## Data Availability

The data that support the findings of this study are available from the corresponding author upon reasonable request.
